# Evidence of nerve hypertrophy in patients with inclusion body myositis on lower limb MRI

**DOI:** 10.1002/mus.27728

**Published:** 2022-10-07

**Authors:** Mostafa Elmansy, Jasper M. Morrow, Sachit Shah, Arne Fischmann, Stephen Wastling, Mary M. Reilly, Michael G. Hanna, Eman Mohamed Helmy, Saleh Saleh El‐Essawy, John S. Thornton, Tarek A. Yousry

**Affiliations:** ^1^ Neuroradiological Academic Unit UCL Queen Square Institute of Neurology London UK; ^2^ Department of Radiology Mansoura University Hospitals Mansoura Egypt; ^3^ Queen Square Centre for Neuromuscular Diseases, Department of Neuromuscular Diseases UCL Queen Square Institute of Neurology London UK; ^4^ Institute of Radiology and Nuclear Medicine and Breast Center St. Anna Hirslanden Klinik St. Anna Lucerne Switzerland

**Keywords:** Charcot–Marie–Tooth disease, inclusion body myositis, magnetic resonance imaging, magnetic resonance neurography, nerve enlargement

## Abstract

**Introduction/aims:**

Inclusion body myositis (IBM) is a myopathic condition but in some patients has been associated with an axonal length‐dependent polyneuropathy. In this study, we quantified the cross‐sectional area of the sciatic and tibial nerves in patients with IBM comparing with Charcot–Marie–Tooth disease type 1A (CMT1A) and healthy controls using magnetic resonance neurography (MRN).

**Methods:**

MRN of the sciatic and tibial nerves was performed at 3T using MPRAGE and Dixon acquisitions. Nerve cross‐sectional area (CSA) was measured at the mid‐thigh and upper third calf regions by an observer blinded to the diagnosis. Correlations were performed between these measurements and clinical data.

**Results:**

A total of 20 patients with IBM, 20 CMT1A and 29 healthy controls (age‐ and sex‐matched) were studied. Sciatic nerve CSA was significantly enlarged in patients with IBM and CMT1A compared to controls (sciatic nerve mean CSA 62.3 ± 22.9 mm^2^ (IBM) vs. 35.5 ± 9.9 mm^2^ (controls), *p* < 0.001; and 96.9 ± 35.5 mm^2^ (CMT1A) vs. 35.5 ± 9.9 mm^2^ (controls); *p* < 0.001). Tibial nerve CSA was also enlarged in IBM and CMT1 patients compared to controls.

**Discussion:**

MRN reveals significant hypertrophy of the sciatic and tibial nerves in patients with IBM and CMT1A compared to controls. Further studies are needed to correlate with neurophysiological measures and assess whether this finding is useful diagnostically.

## 1 INTRODUCTION

Inclusion body myositis (IBM) presents with weakness of the quadriceps and the forearm flexor muscles associated with mixed inflammatory and degenerative features on muscle biopsy.^1^, ^2^ Enlargement of peripheral nerves is a feature of some inherited and acquired neuropathies such as Charcot–Marie–Tooth disease (CMT)^3^, ^4^ but is not a feature expected in patients with myopathy. There is controversy over the possibility of concurrent nerve involvement in IBM patients. Although not a dominant feature clinically, abnormalities in conventional nerve conduction studies have been demonstrated in up to 35% of patients.^5^, ^6^, ^7^ Electromyography in IBM yields a similarly mixed picture of both patterns of large amplitude, long duration neurogenic motor unit potentials and short duration, lower amplitude myopathic motor unit potentials in myopathies. This is thought to be due to fiber splitting and subsequent denervation of part of the muscle fiber disconnected from the endplate. This mixed picture may contribute to misdiagnosis.^7^, ^8^ MRI of nerves in IBM has not been previously studied.

During muscle segmentation performed in a previous quantitative muscle MRI study in IBM and CMT1A,^9^ a region of interest was defined on the sciatic and tibial nerves, as nerve enlargement is a recognized feature in CMT1A. Because the analysis was performed blinded to diagnosis, all subjects, including IBM patients, were included. A significant increase in tibial and sciatic nerve size was co‐incidentally noted in the IBM patients, and hence the more systematic analysis reported here was performed to delineate this further. The aim of this study was to examine the size of the sciatic and tibial nerves in IBM using magnetic resonance neurography (MRN), perform correlations between these measurements and demographic data as well as clinical disease scores, and to compare these data to CMT1A patients (in whom nerve enlargement is well‐recognized) and healthy controls.

## 2 METHODS

### 2.1 Study design and populations

The imaging data were acquired in a prospective cross‐sectional study at the UCL Institute of Neurology, Queen Square, London. Patients with pathologically or clinically definite IBM according to MRC criteria^1^ and genetically confirmed CMT type 1A patients were recruited between January 19, 2010, and July 7, 2011. That study established the value of quantitative muscle MRI to monitor progression in neuromuscular diseases.^9^

This study was approved by the local ethics committee and all participants provided written informed consent.

### 2.2 MRI examination and analysis

MRN was performed at 3T (Magnetom Trio, Siemens, Erlangen, Germany). Images were acquired using a combination of the in‐built spine‐matrix call, a body flex coil, and a “PA matrix” lower leg receive coil. To evaluate the sciatic and tibial nerves, CSA, axial, and coronal plane reformatted volumetric T1‐weighted magnetization‐prepared rapid gradient echo (MPRAGE) images of the thigh were examined (1 mm isotropic resolution), in addition to axial 2D Dixon images (out‐of‐phase fat‐water magnitude images with a TE of 3.45 ms), which were acquired at both thigh and calf level regions with slice gap 20 mm. The acquisition protocols are detailed in Supplemental Table 1, and example images presented in Supplemental Figure 1.

#### 2.2.1 Software and image processing

The sciatic nerve was manually segmented, separately for left and right lower‐limbs, by a radiologist (M.E.) blinded to the diagnosis and the clinical data using ITK‐SNAP software.^10^ The nerve was identified and segmented with a single slice free‐hand multivoxel region of interest (ROI) at the mid‐thigh region with as reference‐point the adductor longus (AL) muscle insertion at both MPRAGE and 2D Dixon Supplemental Figure 1(A–F). The tibial nerve was segmented at 2D Dixon only using the same technique in the upper third of the calf with as reference landmark the point where the flexor halluics longus tendon (FHL) wraps around the fibular head just anteriorly to the soleus sling Supplemental Figure 1(G–I). The CSA of each nerve was extracted.

#### 2.2.2 Clinical assessment

Clinical data collected from IBM, CMT1A, and control participants included age, weight, and height, in addition to the age at symptom onset and duration in the disease groups. The clinical IBM and CMT1A disease scores recorded included the total quality of life score SF36 and the sum of lower limb MRC grades (MRC‐LL). Additionally, functional rating scales were calculated using the inclusion body myositis functional rating scale (IBMFRS) and inclusion body myositis functional rating scale lower limb (IBMFRS‐LL)^11^ for IBM and the CMT examination score (CMTES)^12^ for CMT1A. The clinical and epidemiological data were correlated with MRI sciatic and tibial nerve measurements.

#### 2.2.3 Statistical analysis

Statistical analysis was performed using SPSS (IBM, Armonk, NY). Independent T‐tests were used to compare mean nerve CSAs between groups, and our nerve measurements were normally distributed and expressed in mean ± SD. Spearman's correlation was used to test the correlation between the MPRAGE and Dixon thigh level CSA measurements, as well as the correlation between nerve CSAs, and the clinical data. Strong or very strong correlations were defined as those with coefficients of 0.70–1.00, moderate correlations as 0.40–0.69, and weak as 0.10–0.39.^13^

## 3 RESULTS

Briefly, the study included 20 IBM patients (mean age ± SD, 66.9 ± 8.8 y), 20 CMT type 1A (mean age ± SD, 46.6 ± 14.5), and 29 age and gender‐matched healthy controls (mean age ± SD, 53.5 ± 16.9 y). In order to match the IBM patients' age, we subcategorized our healthy controls into two groups, below and ≥50 y old, and performed additional analysis for the healthy control group ≥50 y (n = 19).

One CMT1A patient had missing calf 2D Dixon images, and one healthy control was missing thigh 2D Dixon data. Thigh MPRAGE‐Neurography was available for 18 IBM patients, 18 CMT1A patients, and 27 healthy controls (Table 1).

**TABLE 1:** Cross‐sectional area measurements for the sciatic nerve (2D Dixon & MPRAGE) and tibial nerve (2D Dixon) in our groups

Sciatic nerve 2D Dixon	IBM	Controls all	Control < 50 y	Control ≥ 50 y
	n = 20	n = 29	n = 10	n = 19
Right	63 ± 19.9***	38.7 ± 15.5	31.2 ± 7.1	42.6 ± 17.4*
Left	59.3 ± 22.7***	37.7 ± 11.7	29 ± 5.2	42.2 ± 11.7**

Sciatic nerve MPRAGE	IBM	Controls all	Control < 50 y	Control ≥ 50 y
	n = 18	n = 27	n = 10	n = 19
Right	63.7 ± 21.5***	35.7 ± 10.2	29.4 ± 5.7	38.9 ± 10.5**
Left	60.9 ± 24.5***	35.4 ± 9.7	30.5 ± 7	37.8 ± 10

Tibial nerve 2D Dixon	IBM	Controls all	Control < 50 y	Control ≥ 50 y
	n = 20	n = 28	n = 10	n = 19
Right	15 ± 5.2 ***	8.9 ± 3.4	8.9 ± 3.3	8.8 ± 3.5
Left	16.8 ± 6 ***	9.8 ± 3.4	8.9 ± 3.3	10.3 ± 3.4

*Note*: All measurements are in mm^2^ and are given as mean ± SD. *p*‐values IBM/CMT1A versus control, or control ≥50 y versus <50 y: *** <0.001, ** <0.01, *p < 0.05.

**FIGURE 1:**
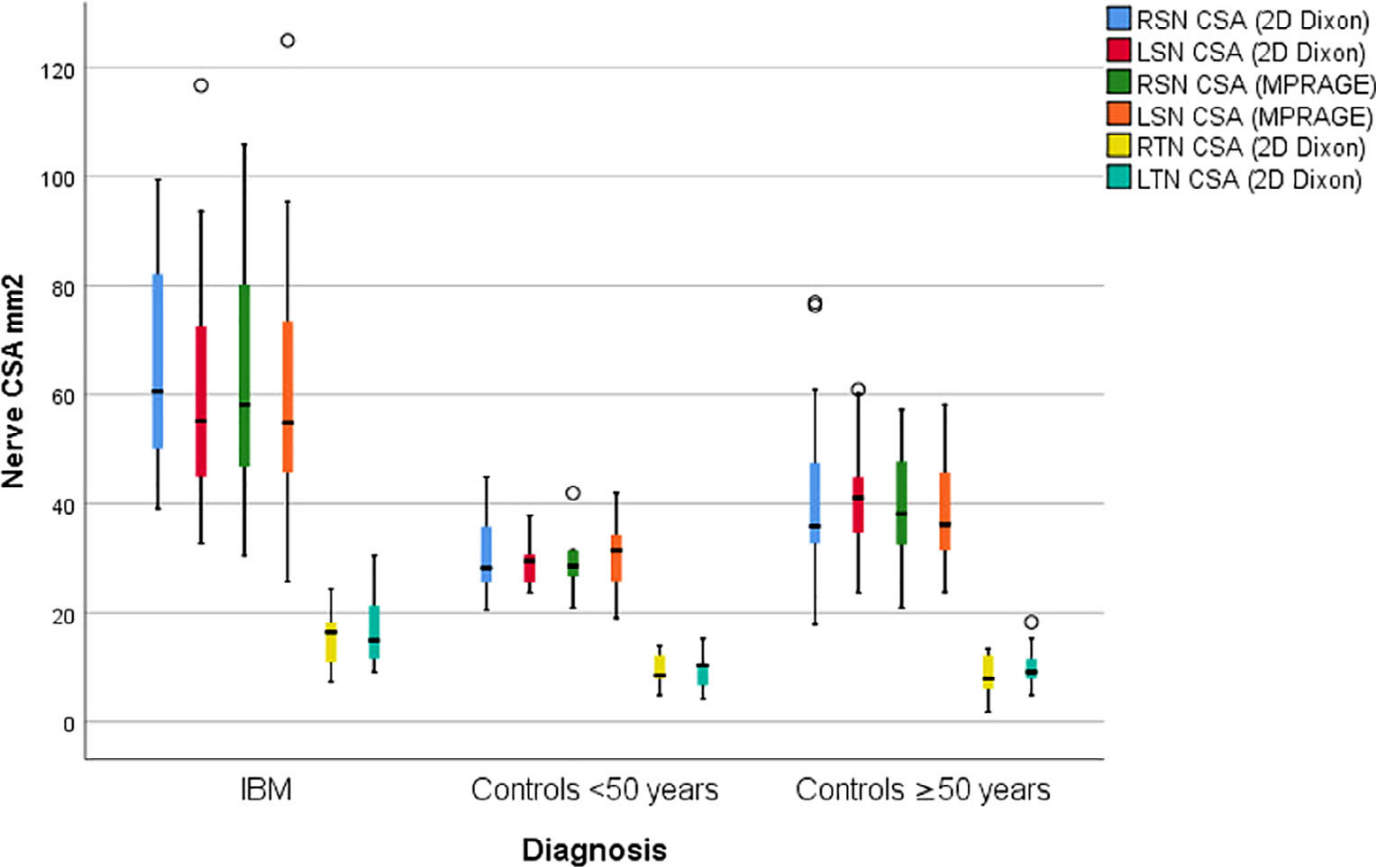
Boxplot showing nerve CSA measurements in IBM versus healthy control groups. LSN, left sciatic nerve; LTN, left tibial nerve; RSN, right sciatic nerve; RTN, right tibial nerve

Mean sciatic and tibial nerve CSA was more than 50% greater in the IBM group versus controls (Table 1, Figure 1). There was no difference in the sciatic and tibial nerve measurements between left and right sides (*p*‐values: 0.3–0.9). As expected, sciatic and tibial nerve CSA was markedly increased in CMT1A patients, much greater than in IBM patients and controls with all comparisons highly significant (*p* < 0.001).

**FIGURE 2:**
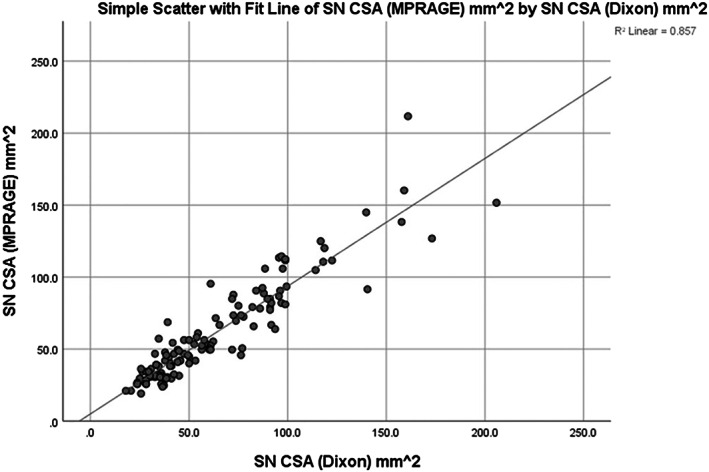
The correlation between the CSA measurements of the sciatic nerve on MPRAGE and 2D Dixon

Mean sciatic nerve CSA measurements were similar between MPRAGE and 2D Dixon methods with a strong correlation between these measurements (*r*_s_ = 0.92 at *p*‐value < 0.001) (Figure 2).

- Correlation with the clinical and epidemiological data for IBM:
  There were no significant positive correlations between the sciatic and tibial nerve CSAs and age, the clinical scores IBMFRS/IBMFRS‐LL or age at onset and duration of the disease.
- Correlation with the clinical and epidemiological data for CMT1A:
  Unlike the IBM patients, there were statistically significant positive moderate correlations between the sciatic nerve measurements and both age (2D Dixon *r*_s_ = 0.54, *p* = 0.01; MPRAGE *r*_s_ = 0.52, *p* = 0.01) and duration of the disease (2D Dixon *r*_s_ = 0.56, *p* = 0.01, MPRAGE *r*_s_ = 0.61, *p* < 0.01). However, there were no significant correlations noted between sciatic and tibial nerve measurements and the clinical scores.
- Correlation between the age of controls and sciatic nerve measurements:
  There was a positive moderate correlation between the age of our healthy controls and sciatic nerve measurements (*r*_s_ = 0.43–0.57 at *p*‐value<0.01). Sciatic but not tibial nerve size was greater in older controls (≥50 y) but were still less than the age matched IBM group. Similarly, the older group of healthy volunteers ≥50 y had a larger mean CSA of the sciatic nerve (42.4 ± 14.5 mm^2^) than the younger group (30.1 ± 6.1 mm^2^) (Table 1, Figure 1).

## 4 DISCUSSION

This study showed significantly increased cross‐sectional areas of the sciatic and tibial nerves on MRI in IBM patients compared with age‐matched healthy controls and confirms markedly enlarged peripheral nerves in CMT1A patients. A significant positive correlation between sciatic nerve size and age was seen in both the CMT1A and healthy control groups but not in IBM patients.

The increase in nerve size in the IBM patients was less than that seen in CMT1A patients, but was consistent across MPRAGE and 2D Dixon measurements in the sciatic nerve and 2D Dixon measurements of the tibial nerve. Although unlikely to be considered a primary method for diagnosis in IBM, the pattern of muscle imaging is of diagnostic value in IBM^14^ and if a radiologist notes nerve enlargement on a scan of a patient with myopathy, this may raise the likelihood of IBM as a diagnosis. However, to be diagnostically useful, this difference would need to be validated by comparing nerve size in IBM with patients with other myopathies, to ensure nerve enlargement is not associated with myopathy more generally.

We also observed a moderate positive correlation between sciatic nerve size and age in healthy controls resulting in a mean difference of about 10 mm^2^ between our controls younger and greater than 50 y of age. This positive correlation has been similarly previously reported with MRI.^15^ An ultrasound study^16^ also showed a correlation of sciatic (*r* = 0.27, *p* = 0.04) but not tibial nerve size with age, consistent with our results.

The IBM patients had increased neve size compared to the age‐matched controls and had enlargement of both sciatic and tibial nerves, so the effect seen is not simply due to aging. However, we did not find a significant correlation between the nerve measurements and the clinical or demographic data, even with age, as we found in healthy controls and CMT1A. The latter might be explained by the more limited, older age range in the IBM patients than the CMT1 and control groups.

Histopathological studies provide evidence of concurrent axonal length‐dependent polyneuropathy in a proportion of IBM patients. Both intramuscular and sural nerve studies have demonstrated myelinated fiber axonal loss and Wallerian degeneration and numerous changes in Schwann cells and myelin sheaths in IBM patients on both conventional and electron microscopy.^6^, ^17^ However, these nerve biopsies were performed on distal sural nerves, so the pathological basis of any nerve changes in the more proximal nerves such as those imaged in this study is unknown.

There were some limitations and potential unintended bias in our study related to nerve segmentation due to limb length variations, differential aggressive muscle atrophy in the diseased groups, and also partial volume artifact in 2D Dixon images. Therefore, in some cases, we had to change the point of standardization for one slice‐level up/down. However, we think this is unlikely to impact our results and conclusions. It was not possible to delineate the tibial nerve on the MPRAGE sequence alone. The smaller size of the nerve and the derivation of the MPRAGE sequence from T1 limit the visualization of the nerve signal and differentiation between it and the posterior tibial vascular bundle. This was not problematic in the segmentation of the sciatic nerve as it is of considerable size and relatively distant posteriorly from the vascular femoral vessels. Finally, we did not study the quantitative neurography biomarkers including T2 relaxometry and proton spin density, and we did not correlate our nerve measurements with nerve conduction studies. Future studies with neurophysiology and longitudinal follow‐up studies are needed to determine whether nerve CSA is useful in monitoring disease progression.

## 5 CONCLUSION

This study confirmed sciatic and tibial nerve enlargement in IBM patients on MRI using comparators of CMT1A and healthy controls. Our study did not find significant correlations between the IBM clinical data and disease scores with nerve CSA. Further studies are needed to validate the diagnostic value of nerve hypertrophy in IBM and to better understand the underlying pathogenic mechanisms.

**LIST OF ABBREVIATIONS**
AL: adductor longus muscle
AUC: area under the curve
CMT1A: Charcot–Marie‐Tooth type 1A
CMTES: Charcot–Marie‐Tooth examination score
CSA: cross‐sectional area
FHL: flexor halluics longus muscle
FOV: field of view
IBM: inclusion body myositis
IBM‐FRS/IBM‐FRSLL: inclusion body myositis functional rating scale lower limb
MPRAGE: magnetization‐prepared rapid gradient echo
MRC: medical research council
MRC‐LL: medical research council sum of muscle grades of lower limbs
MRI: magnetic resonance imaging
MRN: magnetic resonance neurography
NCS: nerve conduction velocity
Rs: Spearman correlation
ROI: region of interest
SN: sciatic nerve
T: tesla
TN: tibial nerve
US: ultrasound

## AUTHOR CONTRIBUTIONS

All authors have read and approved the manuscript.

- The study concept and design were proposed by M.E. and J.M.
- Statistical analysis of data: S.W., S.E., and E.H.
- Writing the original manuscript: M.E.
- Revision of the manuscript for important intellectual content: J.M., S.S., A.F., M.R., M.H., J.T., T.Y.

## CONFLICTS OF INTEREST

None of the authors has any conflict of interest to disclose.

## ETHICS STATEMENT

We confirm that we have read the Journal's position on issues involved in ethical publication and affirm that this report is consistent with those guidelines. The study was approved by the local ethics committee and all participants provided written informed consent.

## Supporting information


**Supplemental Table 1.** Our MRI protocol and sequences.

**Supplemental Figure 1.** (A–C) MRI MPRAGE thigh images for CMT1A, IBM, and healthy control respectively. (D–F) 2D Dixon thigh images for the same groups. (G–I) 2D Dixon calf images for the same groups. Sciatic nerve (arrowed) segmented at the mid‐thigh region (A–F). Adductor longus (arrowhead) (C&F) (Reference landmark for sciatic nerve measurements). Tibial nerve (dash arrow) (G–I) segmented at the upper calf region. Flexor hallucis longus (curved arrow) (I) (Reference standard point for tibial nerve CSA measurements on 2D Dixon).

**Supplemental Figure 2.** Receiver operating characteristic (ROC) curve for SN; sciatic nerve (A) and TN; tibial nerve (B) CSA; cross‐sectional area on 2D Dixon in IBM versus CMT1A. AUC; area under the curve.

## Data Availability

The data that support the findings of this study are available on request from the corresponding author. The data are not publicly available due to privacy or ethical restrictions.
