# Supplementary material for: Evidence of nerve hypertrophy in patients with inclusion body myositis on lower limb MRI
Source: Muscle Nerve. 2022 Oct 7;66(6):744–9. doi: 10.1002/mus.27728 (PMC10286743; doi:10.1002/mus.27728)
Supplement: Supplementary file 1 — Supplemental Table 1. Our MRI protocol and sequences. [file MUS-66-744-s002.docx]

**Supplemental table (1)** Our MRI protocol and sequences.

| **MRI** | **Slice thickness/gap**  **(mm)** | **TR/TE**  **(ms)** | **Field of View (FOV) (cm^2^)** | **Base resolution**  **(pixels)** | **In plane resolution (mm)** | **Inversion time (ms)** | **Flip angle**  **(degrees)** |
| --- | --- | --- | --- | --- | --- | --- | --- |
| **MPRAGE** | 1/0 | 1680/4.41 | 50.0x20.3 | 512x208 | 0.97 x 0.97 | 820 | 15 |
| **2D Dixon** | 10/10 | 100/3.45 | 44x20.6 | 512x240 | 0.86 x 0.86 | N/A | 10 |
