# Supplementary figures and images for: Evidence of nerve hypertrophy in patients with inclusion body myositis on lower limb MRI
Source: Muscle Nerve. 2022 Oct 7;66(6):744–9. doi: 10.1002/mus.27728 (PMC10286743; doi:10.1002/mus.27728)

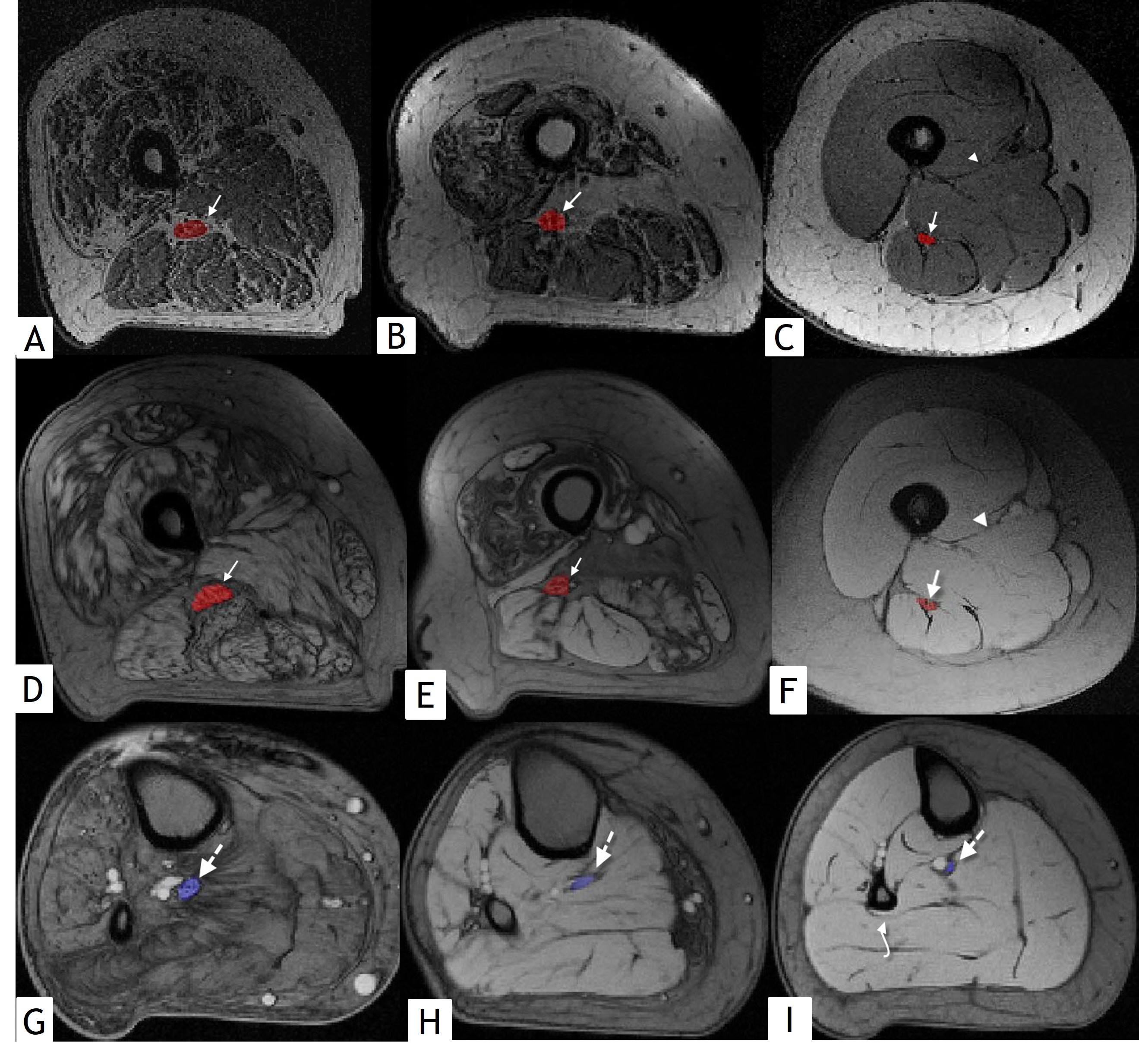

Supplement: Supplementary file 2 — Supplemental Figure 1. (A–C) MRI MPRAGE thigh images for CMT1A, IBM, and healthy control respectively. (D–F) 2D Dixon thigh images for the same groups. (G–I) 2D Dixon calf images for the same groups. Sciatic nerve (arrowed) segmented at the mid‐thigh region (A–F). Adductor longus (arrowhead) (C&F) (Reference landmark for sciatic nerve measurements). Tibial nerve (dash arrow) (G–I) segmented at the upper calf region. Flexor hallucis longus (curved arrow) (I) (Reference standard point for tibial nerve CSA measurements on 2D Dixon). [file MUS-66-744-s003.jpg]

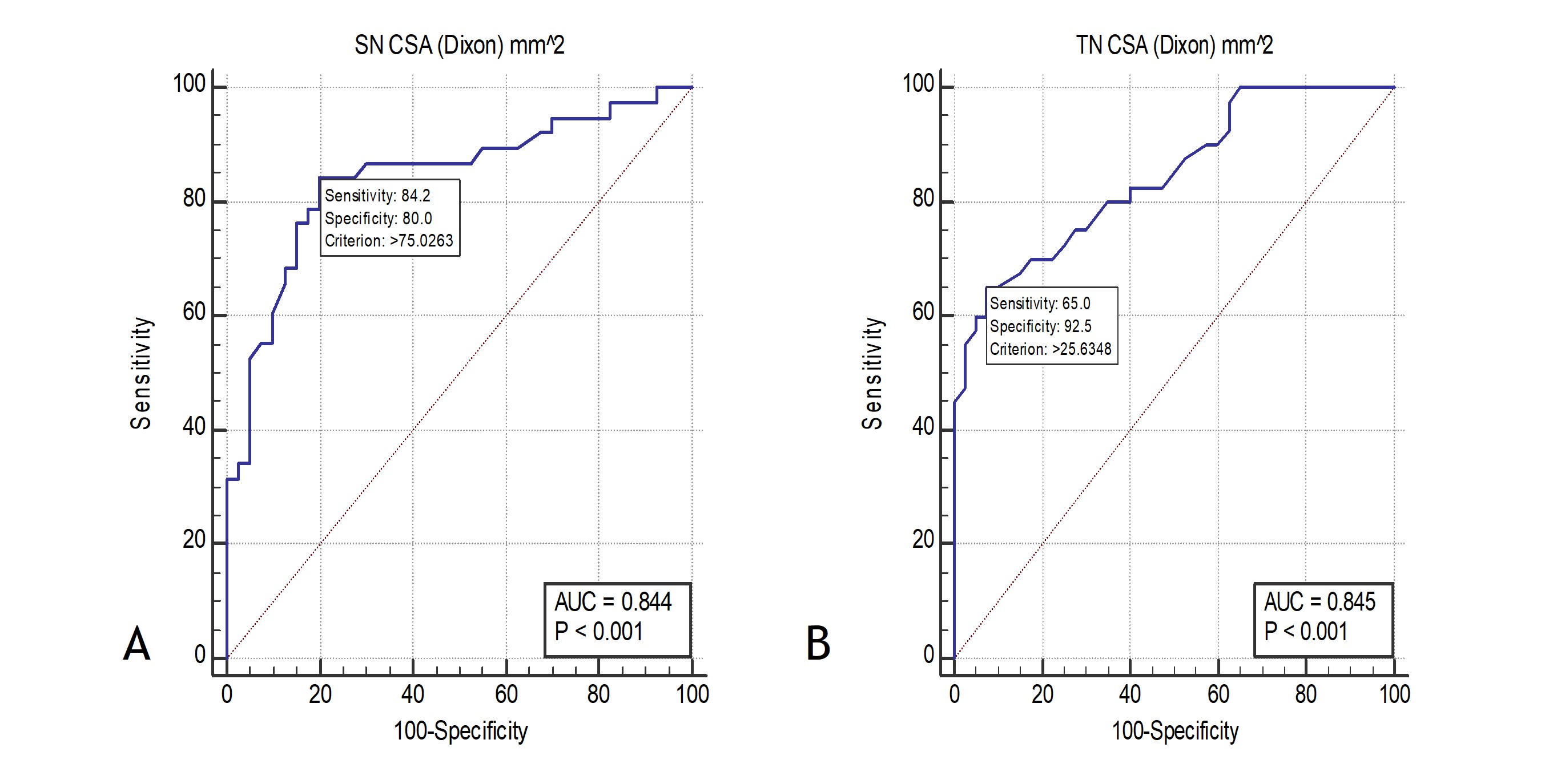

Supplement: Supplementary file 3 — Supplemental Figure 2. Receiver operating characteristic (ROC) curve for SN; sciatic nerve (A) and TN; tibial nerve (B) CSA; cross‐sectional area on 2D Dixon in IBM versus CMT1A. AUC; area under the curve. [file MUS-66-744-s001.jpg]
